# Supplementary material for: Physical Activity and the Development of Post-Transplant Diabetes Mellitus, and Cardiovascular- and All-Cause Mortality in Renal Transplant Recipients
Source: J Clin Med. 2020 Feb 3;9(2):415. doi: 10.3390/jcm9020415 (PMC7074375; doi:10.3390/jcm9020415)
Supplement: Supplementary file 1 [file jcm-09-00415-s001.pdf]

Journal of Clinical Medicine

SUPPLEMENTARY MATERIALS

Contents	
Table S1	Model 7 and separate adjustments for BMI and waist circumference in the associations of MVPA of with long-term health outcomes in RTRs
Table S2.	Baseline characteristics of RTRs according to the presence of long-term health outcomes
Figure S1.	Level of daily-life PA according to occupational status (A) and age (B).

Table S1. Model 7 and separate adjustments for BMI and waist circumference in the associations of MVPA of with long-term health outcomes in RTRs

**Physical**	**MVPA (cont.)**		**No-MVPA**	**MVPA-1**		**MVPA-2**	
**activity**	HR (95% CI)	*P*	(Ref)	HR (95% CI)	*P*	HR (95% CI)	*P*
Post-transplant DM							
Model 7	0.88 (0.79-0.99)	0.03	1.00	0.63 (0.31-1.25)	0.19	0.50 (0.25-1.03)	0.06
Model 7A	0.90 (0.80-0.99)	0.043	1.00	0.63 (0.32-1.25)	0.19	0.55 (0.27-1.10)	0.09
Model 7B	0.89 (0.80-0.99)	0.03	1.00	0.64 (0.32-1287)	0.21	0.52 (0.26-1.06)	0.07
Cardiovascular mortality							
No. of events	53/589		26	14		13	
Model 7	0.85 (0.75-0.96)	0.01	1.00	0.51 (0.23-1.11)	0.09	0.40 (0.17-0.92)	0.03
Model 7A	0.84 (0.74-0.94)	0.001	1.00	0.46 (0.23-0.96)	0.04	0.35 (0.15-0795)	0.01
Model 7B	0.85 (0.75-0.97)	0.01	1.00	0.51 (0.24-1.18)	0.09	0.40 (0.17-0.39)	0.03
All-cause mortality							
No. of events	129/650		76	27		26	
Model 7	0.84 (0.78-0.89)	<0.001	1.00	0.40 (0.25-0.63)	<0.001	0.37 (0.23-0.59)	<0.001
Model 7A	0.83 (0.78-0.89)	<0.001	1.00	0.39 (0.25-0.61)	<0.001	0.37 (0.24-0.58)	<0.001
Model 7B	0.84 (0.78-0.89)	<0.001	1.00	0.40 (0.25-0.64)	<0.001	0.38 (0.24-0.60)	<0.001

DM=Diabetes mellitus, MVPA=moderate-to-vigorous physical activity.

Model 7: model 1 + adjustment for BMI and waist circumference

Model 7A: model 1 + adjustment for BMI

Model 7B: model 1 + adjustment for waist circumference

Table S2. Baseline characteristics of RTRs according to the presence of long-term health outcomes

**Variables**	**Post-transplant diabetes mellitus**		**All-cause mortality**	
	Without (n=521)	With (n=129)	Without (n=449)	With (n=50)
General characteristics				
Age (years)	51.5 ± 13.4	52.7 ± 10.9	50.9 ± 12.5	60.5 ± 10.5*

Male gender (%, n)	56.7 (245)	62.3 (33)	56.1 (311)	59.2 (90)
Current smoking (%, n)	12.0 (52)	20.8 (11)	11.9 (66)	11.8 (18)
Alcohol use (g/day)	3.1 (0-12.4)	3.5 (0-10.0)	3.2 (0.0-11.6)	1.0 (0-6.5)*
Total energy intake (kcal/d)	2203.1 ± 658.4	2205.9 ± 522.3	2203.2 ± 656.1	2035.1 ± 554.6*
SBP (mm Hg)	134.9± 17.0	141.4 ± 17.6*	135.4 ± 17.1	138.0 ± 18.9
DBP (mm Hg)	82.6 ± 10.9	87.0 ± 11.1*	82.9 ± 11.1	81.0 ± 10.8
BMI (kg/m^2)	25.8 ± 4.7	27.6 ± 4.7	26.5 ± 4.7	27.2 ± 5.1
Waist in men (cm)	98.4 ± 11.7	106.6 ± 13.6	100.3 ± 12.7	104.8 ± 15.6
Waist in women (cm)	92.1 ± 15.6	99.6 ± 16.2	94.1 ± 15.6	98.6 ± 14.8*
Total cholesterol (mmol/L)	5.1 ± 1.1	5.3 ± 1.3	5.1 ± 1.1	5.1 ± 1.3
Triglyceride (mmol/L)	1.6 (1.2-2.1)	2.2 (1.4-3.1)*	1.7 (1.2-2.3)	1.9 (1.4-2.6)*
HDL-C in men (mmol/L)	1.3 ± 0.4	1.1 ± 0.3*	1.3 ± 0.4	1.2 ± 0.4
HDL-C in women (mmol/L)	1.6 ± 0.5	1.4 ± 0.3*	1.6 ± 0.5	1.4 ± 0.4*
Kidney function				
eGFR (mL/min/1.73m^2)	53.0 ± 20.5	51.0 ± 16.8	54.0 ± 20.4	46.2 ± 19.1
Albumin excretion (mg/24h)	269.1 ± 709.2	235.4 ± 625.0	230.1 ± 681.8	410.7 ± 833.7*
Proteinuria (%, n)	20.4 (88)	20.8 (11)	19.0 (105)	34.9 (53)*
Primary renal disease (%, n)				
Glomerulosclerosis	28.9 (125)	34.0 (18)*	29.8 (165)	21.7 (33)
Glomerulonephritis	7.9 (34)	7.5 (4)	8.5 (47)	4.6 (7)
Tubulointerstitial nephritis	12.5 (54)	11.3 (6)	12.6 (70)	9.2 (14)
Polycystic kidney disease	22.5 (97)	17.0 (9)	19.9 (110)	24.3 (37)
Renal hypodysplasia	3.7 (16)	3.8 (2)	4.2 (23)	3.9 (6)
Renavascular diseases	5.1 (22)	5.7 (3)	5.6 (31)	5.9 (9)
Diabetes mellitus	-	-	2.9 (16)	13.2 (20)*
Others	17.6 (76)	17.0 (9)	16.6 (92)	17.1 (26)
Transplant characteristics				
Transplant vintage (months)	15.0 (2.0-44.0)	10.0 (1.0-34.0)	14.0 (2.0-41.0)	19.0 (0-39.0)
Living donor (%, n)	35.2 (152)	37.7 (20)	39.2 (217)	16.4 (25)*
Pre-emptive transplant (%, n)	17.4 (75)	15.1 (8)	18.1 (100)	8.6 (13)*
Cold ischemia time (h)	15.2 (2.7-21.3)	15.5 (2.6-24.0)	14.5 (2.6-21.0)	18.0 (2.7-24.0)*
Acute rejection	24.8 (107)	22.6 (12)	25.8 (143)	30.3 (46)
Calcineurin inhibitor				
Cyclosproine (%, n)	36.8 (159)	49.1 (26)	38.4 (213)	42.1 (64)
Tacrolimus (%, n)	17.1 (74)	20.8 (11)	18.1 (100)	19.7 (30)
Proliferation inhibitor				
Azathioprine (%, n)	19.0 (82)	15.1 (8)	17.5 (97)	17.1 (26)
Mycophenolate (%, n)	66.7 (288)	60.4 (32)	67.9 (376)	57.2 (87)*
Prednisolone dose (mg)	10.0 (7.5-10.0)	10.0 (7.5-10.0)	10.0 (7.5-10.0)	10.0 (7.5-10.0)

Data are presented as mean ± SD or median (interquartile range) and percentage (number). SBP, systolic blood pressure; DBP, diastolic blood pressure; BMI, body mass index; HDL-C, high-density lipoprotein cholesterol; eGFR, estimated

glomerular filtration rate. Differences were tested by analysis of variance or Kruskal-Wallis for continuous variables and with $\chi 2$ test for categorical variables.

* p<0.05.

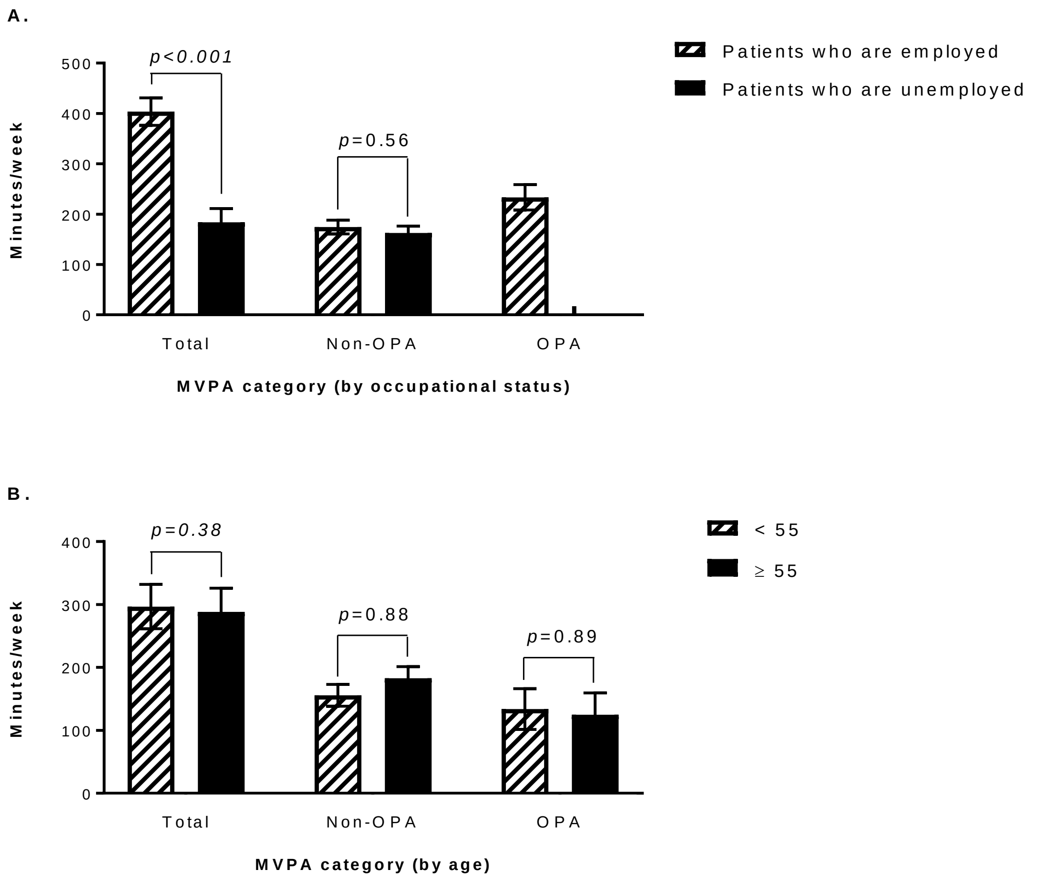

Figure S1. Level of daily-life PA according to occupational status (A) and age (B).
